# Defining the impact of flavivirus envelope protein glycosylation site mutations on sensitivity to broadly neutralizing antibodies

**DOI:** 10.1128/mbio.03048-23

**Published:** 2024-01-09

**Authors:** Maya Contreras, Jackson B. Stuart, Lisa M. Levoir, Laura Belmont, Leslie Goo

**Affiliations:** 1 Vaccine and Infectious Disease Division, Fred Hutchinson Cancer Center, Seattle, Washington, USA; 2 Graduate Program in Pathobiology, Department of Global Health, University of Washington, Seattle, Washington, USA; 3 Molecular and Cellular Biology Graduate Program, University of Washington, Seattle, Washington, USA; University of Colorado School of Medicine, Aurora, Colorado, USA

**Keywords:** flavivirus, broadly neutralizing antibodies, envelope glycosylation

## Abstract

Antibodies targeting an envelope dimer epitope (EDE) cross-neutralize Zika virus (ZIKV) and dengue virus (DENV) and have thus inspired an epitope-focused vaccine design. There are two EDE antibody subclasses (EDE1, EDE2) distinguished by their dependence on viral envelope protein *N*-linked glycosylation at position N153 (DENV) or N154 (ZIKV) for binding. Here, we determined how envelope glycosylation site mutations affect *neutralization* by EDE and other broadly neutralizing antibodies. Consistent with structural studies, mutations abolishing the N153/N154 glycosylation site increased DENV and ZIKV sensitivity to neutralization by EDE1 antibodies. Surprisingly, despite their location at predicted contact sites, these mutations also increased sensitivity to EDE2 antibodies. Moreover, despite preserving the glycosylation site motif (N-X-S/T), substituting the threonine at ZIKV envelope residue 156 with a serine resulted in loss of glycan occupancy accompanied with increased neutralization sensitivity to EDE antibodies. For DENV, the presence of a serine instead of a threonine at envelope residue 155 retained glycan occupancy, but nonetheless increased sensitivity to EDE antibodies, in some cases to a similar extent as mutation at N153, which abolishes glycosylation. Envelope glycosylation site mutations also increased ZIKV and DENV sensitivity to other non-EDE broadly neutralizing antibodies, but had limited effects on ZIKV- or DENV-specific antibodies. Thus, envelope protein glycosylation is context-dependent and modulates the potency of broadly neutralizing antibodies in a manner not predicted by existing structures. Manipulating envelope protein glycosylation could be a novel strategy for engineering vaccine antigens to elicit antibodies that broadly neutralize ZIKV and DENV.

**IMPORTANCE:**

Antibodies that potently cross-neutralize Zika (ZIKV) and dengue (DENV) viruses are attractive to induce via vaccination to protect against these co-circulating flaviviruses. Structural studies have shown that viral envelope protein glycosylation is important for binding by one class of these so-called broadly neutralizing antibodies, but less is known about its effect on neutralization. Here, we investigated how envelope protein glycosylation site mutations impact the potency of broadly neutralizing antibodies against ZIKV and DENV. We found that glycan occupancy was not always predicted by an intact N-X-S/T sequence motif. Moreover, envelope protein glycosylation site mutations alter the potency of broadly neutralizing antibodies in a manner unexpected from their predicted binding mechanism as determined by existing structures. We therefore highlight the complex role and determinants of envelope protein glycosylation that should be considered in the design of vaccine antigens to elicit broadly neutralizing antibodies.

## OBSERVATION

Zika virus (ZIKV) and the four dengue virus serotypes (DENV1–4) are closely related flaviviruses. The “150 loop” region of the flavivirus envelope (E) protein contains a potential *N-*linked glycosylation site (PNGS) at residue N154 or N153 for ZIKV or DENV, respectively. The ZIKV 150 loop is elongated compared to that of other flaviviruses (1, 2). The 150 loop PNGS is important for binding by some E dimer epitope (EDE) antibodies, which cross-neutralize ZIKV and DENV1–4 (3, 4). There are two EDE antibody subclasses, of which EDE2, but not EDE1, requires 150 loop glycosylation for efficient binding (3, 5). How sequence variation at this PNGS impacts the potency of EDE and other broadly neutralizing antibodies (bnAbs) is less defined.

**Fig 1:**
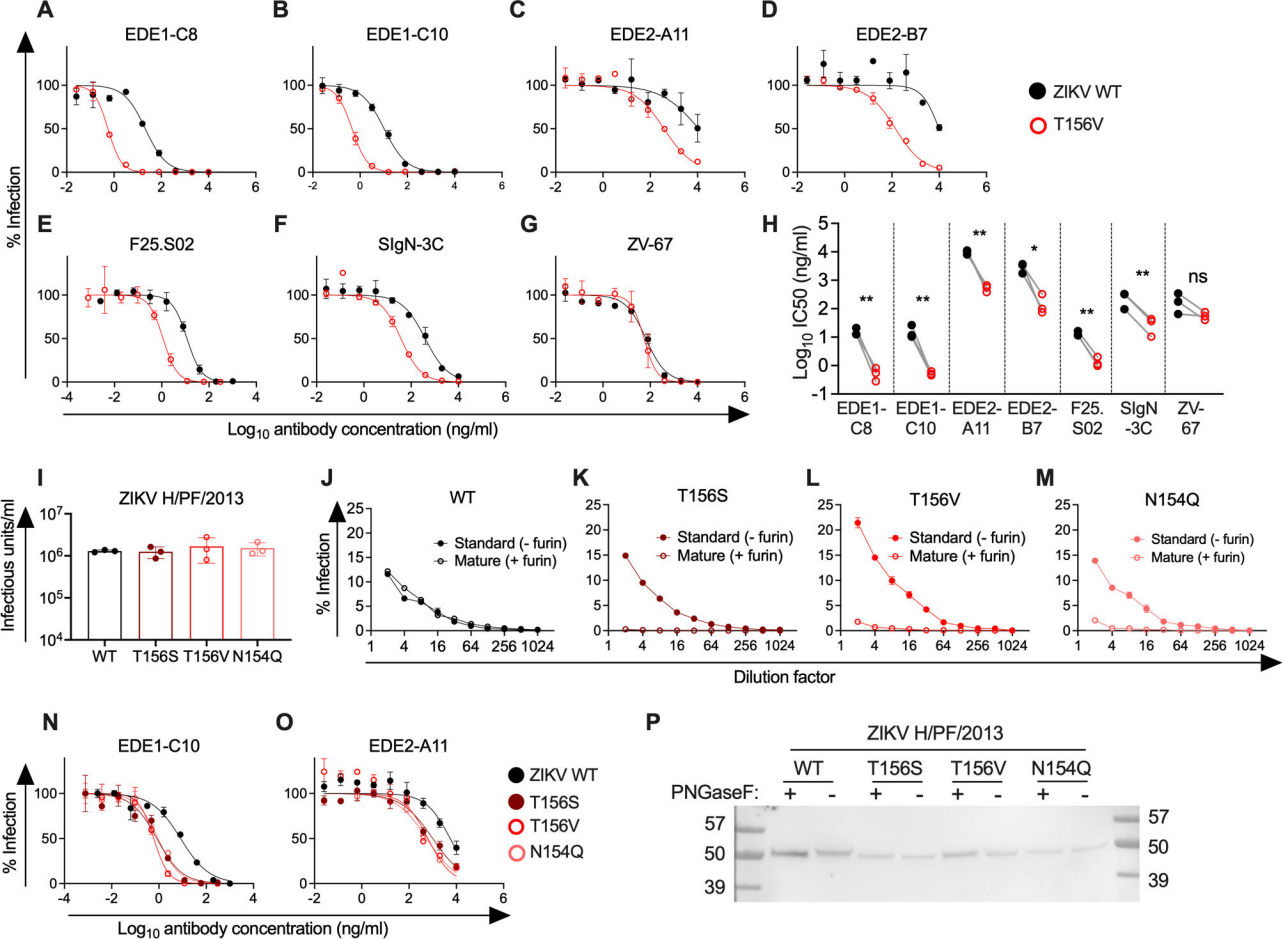
Effect of E protein glycosylation site mutations on ZIKV sensitivity to neutralizing antibodies. (**A–G**) Dose-response neutralization assays using the indicated (**A–F**) bnAbs or (**G**) ZIKV-specific antibody (ZV-67) against ZIKV H/PF/2013 wild-type (WT) (black) or T156V (red) RVPs. Data are representative of three independent experiments, each performed in duplicate wells. Data points and error bars indicate the mean and range of infection in duplicate wells, respectively. (**H**) Antibody IC50 values calculated from three independent experiments in panels A-G in which WT and T156V ZIKV RVPs were tested in parallel. Statistical significance was determined by ratio paired *t*-tests. **, *P* < 0.01; *, *P* < 0.05; ns, not significant. (**I**) Bar graphs show the mean infectious titers of three independent standard preparations of ZIKV RVPs, each represented by a data point. Error bars indicate the standard deviation. (**J–M**) Dose-response infectivity curves of the indicated “standard” or “mature” ZIKV H/PF/2013 WT or mutant RVPs prepared in the absence (filled circles) or presence (open circles) of exogenous furin, respectively. Data shown is from one experiment performed in duplicate wells; error bars indicate the range. (**N and O**) Dose-response neutralization assays using (**N**) EDE1-C10 (**O**) or EDE2-A11 against WT ZIKV H/PF/2013 RVPs or those encoding the indicated E protein mutation. Data are representative of three independent experiments, each performed in duplicate wells. Data points and error bars indicate the mean and range of infection in duplicate wells, respectively. (**P**) E proteins from untreated (−) or PNGaseF-treated (+) RVP lysates were detected by SDS-PAGE and Western blot. Size markers (kDa) are shown in leftmost and rightmost lanes. Data are representative of seven independent experiments performed using two independent WT and mutant RVP stocks prepared in parallel.

To map epitopes of antibodies that neutralize DENV1–4 but not ZIKV, we previously generated a library of DENV2 reporter virus particles (RVPs) (6) in which solvent accessible E protein residues were substituted with those corresponding to the Asian lineage ZIKV strain H/PF/2013 (7). Flavivirus RVPs have been shown to be antigenically similar to non-reporter fully infectious versions (8). A V151T mutation in the DENV2 150 loop reduced the potency of DENV1-4 bnAbs but increased the potency of a control EDE1 bnAb (6). To further investigate how this site impacts EDE potency, we generated RVPs encoding the analogous ZIKV H/PF/2013 E protein T156V mutation based on sequence alignment (Fig. S1A) and observed up to a 32-fold increase in sensitivity to neutralization by EDE1 and EDE2 bnAbs (Fig. 1A through D and H). T156V similarly increased sensitivity to other bnAbs (SIgN-3C and F25.S02) that cross-neutralize DENV1-4 and ZIKV (Fig. 1E and F and H). Unlike EDE antibodies, which target an epitope within the E dimer subunit, the binding footprint of SIgN-3C spans multiple E dimers (9). The F25.S02 epitope has not been structurally defined but mutagenesis studies identified neutralization determinants distinct from those for EDE bnAbs (10). T156V also increased sensitivity to bnAbs by at least eightfold when engineered into the ZIKV MR766 African lineage strain (11) and the Asian lineage PHL/2012 (12), but not THA/2014 (12) strain (maximum of threefold IC50 reduction, Fig. S2). ZIKV THA/2014 is distinguished from other strains tested at two E protein residues (Fig. S1B), which could have compensatory effects. In contrast to our findings with bnAbs, T156V minimally increased sensitivity (average of threefold IC50 reduction) to ZV-67, a ZIKV-specific neutralizing antibody (13) (Fig. 1G and H).

The T156V mutation abrogates the only ZIKV E protein PNGS (Fig. S1A) (1, 14). Increased ZIKV sensitivity to bnAbs conferred by T156V contrasts with the previous observation that abolishing this PNGS did not impact sensitivity to poorly neutralizing antibodies (15). To determine if increased sensitivity of ZIKV T156V to bnAbs is specifically due to loss of the PNGS, we generated ZIKV H/PF/2013 RVP variants containing additional mutations at E residues N154 and T156. Each of these variants, including those abolishing the PNGS, efficiently infected Raji-DCSIGNR cells (Fig. 1I) even though cellular attachment depends on interactions between DCSIGNR and viral glycans (16). As previously suggested, the presence of glycosylated, uncleaved prM retained on the virion surface due to incomplete maturation likely facilitates attachment in the absence of E glycosylation (15). Accordingly, ZIKV RVPs encoding E protein PNGS motif-ablating mutations prepared in the presence of exogenous furin, which improves prM cleavage efficiency, displayed a markedly reduced ability to infect Raji-DCSIGNR cells compared to corresponding virus stocks prepared using standard methods (Fig. 1L and M). In contrast, wild-type (WT) ZIKV prepared with or without excess furin displayed relatively similar infectivity (Fig. 1J). Despite retaining the PNGS motif (N-X-S/T), the infectivity of T156S RVPs prepared with or without furin resembled that of variants that ablate this motif (Fig. 1K).

Like the T156V mutation, the N154Q mutation that disrupts the PNGS increased ZIKV sensitivity to neutralization by EDE1-C10 (Fig. 1N) and EDE2-A11 (Fig. 1O). For EDE1-C10, this finding is consistent with structural studies suggesting that EDE1 antibodies displace the glycan-containing 150 loop to interact with the E protein (5). Surprisingly, despite an intact PNGS motif, ZIKV T156S was also more sensitive to EDE1-C10 and, to a lesser extent, EDE2-A11, compared to WT (Fig. 1N and O). SDS-PAGE and immunoblotting of RVP lysate confirmed that WT ZIKV E protein displayed a slightly lower molecular weight after PNGaseF treatment, indicating glycan cleavage (Fig. 1P). In contrast, ZIKV T156S E protein migration was unaffected by PNGaseF treatment, similar to E protein from ZIKV N154Q or T156V, each of which ablates the PNGS motif. Combined with its infectivity (Fig. 1K) and neutralization profiles (Fig. 1N and O), this finding suggests that the E protein of ZIKV T156S was not glycosylated, although mass spectrometry-based approaches will be needed to assess glycan occupancy at higher resolution. The relative inefficiency of *N*-linked glycosylation associated with the presence of a serine versus a threonine within the PNGS motif (17) has been described for a rabies virus glycoprotein (18).

**Fig 2:**
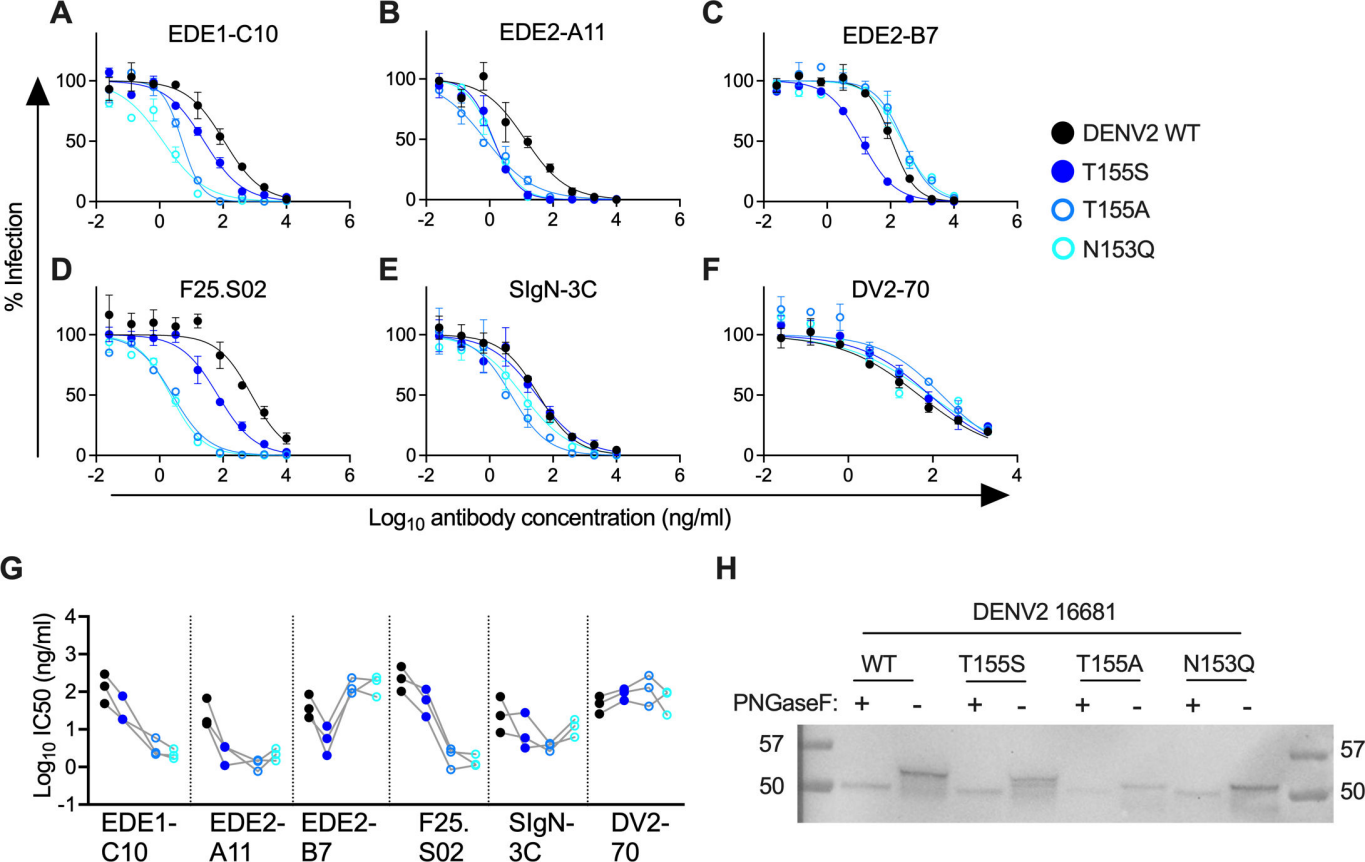
Effect of E protein glycosylation site mutations on DENV2 sensitivity to neutralizing antibodies. (**A–F**) Dose-response neutralization assays using the indicated (**A–E**) bnAbs or (**F**) DENV2-specific antibody (DV2-70) against WT DENV2 16681 RVPs or those encoding the E protein mutation indicated in the key. Data are representative of three independent experiments, each performed in duplicate wells. Data points and error bars indicate mean and range of infection in duplicate wells, respectively. (**G**) IC50 values of antibodies shown on the x-axis from three independent experiments, each indicated by a set of connected data points representing WT or DENV2 16681 RVPs encoding the E protein mutation color coded according to the key in panels A–F. In each experiment, WT and mutant RVPs were tested in parallel. For each antibody, comparisons of IC50 values for mutant versus WT RVPs were not statistically significant (*P* > 0.05, Dunnett’s multiple comparisons test). (**H**) E proteins from untreated (−) or PNGaseF-treated (+) DENV2 RVP lysates were detected by SDS-PAGE and Western blot. Size markers (kDa) are shown in leftmost and rightmost lanes. Data are representative of six independent experiments performed with two independent WT and mutant RVP stocks prepared in parallel. DENV E protein has two PNGS motifs at residues N67 and N153 (Fig. S1A); only mutations that affect the latter are assessed here. The multiple bands observed in untreated wells likely indicate glycosylation at one or both PNGS.

To investigate how DENV 150 loop glycosylation site mutations impact neutralization sensitivity, we generated DENV2 16681 RVP variants encoding mutations at E residues N153 and T155. Unlike our findings with ZIKV, the migration pattern of E protein of DENV2 T155S with or without PNGaseF treatment was similar to that of WT DENV2, suggesting intact glycosylation (Fig. 2H). As expected, E protein from DENV2 encoding the N153Q or T155A mutation, each of which abrogates the PNGS at this position, migrated faster than WT or T155S E protein, indicating loss of glycosylation.

DENV2 mutations that do (T155S) or do not (T155A, N153Q) maintain the 150 loop PNGS motif increased bnAb potency to various extents. Compared to T155A or N153Q, which resulted in a large (100- to 300-fold) increase in sensitivity to EDE1-C10 and F25.S02, T155S increased sensitivity to these bnAbs relatively modestly (4- to 10-fold) (Fig. 2A, D, and G). All three mutations similarly increased sensitivity to EDE2-A11 neutralization by ~20-fold (Fig. 2B and G). The largest effect seen for SIgN-3C was against T155A RVPs (~10-fold increase in sensitivity, Fig. 2E and G). These mutations minimally (<3-fold) impacted the potency of DV2-70, a DENV2-specific antibody (19) (Fig. 2F and G).

The observed increase in neutralization potency associated with the loss of the DENV2 150 loop PNGS was unexpected especially for EDE2-A11, which is predicted to contact the N153 glycan based on structural studies with soluble E protein (5). Mutagenesis of DENV subviral particles also showed that this PNGS was important for EDE2 binding (3). When tested against EDE2-B7, a somatic variant of EDE2-A11 (5), loss of PNGS via N153Q or T155A mutation rendered DENV2 less sensitive to neutralization (Fig. 2C and G), consistent with the EDE2 structural footprint. However, the T155S mutation, which preserves glycosylation, increased DENV2 sensitivity to EDE2-B7 by 124-fold (Fig. 2C and G). Together, these findings demonstrate that the specific amino acid composition within the PNGS motif distinctly impacts DENV2 sensitivity to bnAbs, including those with highly similar structural epitopes and genetic characteristics.

### Conclusions

Flavivirus E protein glycosylation is context-dependent, and sequence variation within the 150 loop PNGS modulates sensitivity to bnAbs in ways not predicted by existing structural studies. We underscore the complex determinants and antigenic consequences of E glycosylation; both are impacted by even a conservative change within the PNGS motif. Notably, there is variation at the PNGS within the 150 loop of currently circulating DENV strains (20). Our findings suggest that mutations within this PNGS are antigenically relevant and should be considered in the design of vaccine antigens to elicit bnAbs.

### Cells

Raji-DCSIGNR cells (a gift from Ted Pierson, NIH) were maintained in RPMI 1640 medium supplemented with GlutaMAX (Cat# 72400-047; ThermoFisher Scientific), 7% fetal bovine serum (FBS) (Cat# 26140079; ThermoFisher Scientific), and 100 U/mL penicillin-streptomycin (Cat# 15140122, ThermoFisher Scientific).

HEK-293T/17 cells (Cat# CRL-11268, ATCC) were maintained in Dulbecco’s modified Eagle’s medium (DMEM, Cat# 11965118; ThermoFisher Scientific) supplemented with 7% FBS and 100 U/mL penicillin-streptomycin. Raji-DCSIGNR and HEK293T/17 cells were generally maintained at 37°C with 5% CO_2_. However, for RVP production, HEK-293T/17 cells were maintained at 30°C in a low-glucose formulation of DMEM (Cat # 12320-032; ThermoFisher Scientific), as previously described (21).

Expi-CHO-S cells (Cat# A29127; ThermoFisher Scientific) were cultured in ExpiCHO Expression Medium (Cat# A2910001; ThermoFisher Scientific) and maintained at 37°C in 8% CO_2_ on a platform rotating at 125 rpm with a rotational diameter of 19 cm.

### Generation of plasmids encoding E protein variants

Previously described plasmids encoding the structural genes (C-prM-E) of DENV2 16681, ZIKV H/PF/2013, ZIKV MR766, ZIKV THA/2014, and ZIKV PHL/2012 (8, 22) (all provided by Ted Pierson, NIH) were used as templates for Q5 site-directed mutagenesis (Cat# E0554S; New England Biolabs) using primers designed with NEBaseChanger (New England Biolabs). Whole plasmids were confirmed by Oxford Nanopore sequencing (Plasmidsaurus) to confirm only the desired mutation was present.

### Production of RVPs

HEK-293T/17 cells were co-transfected with 1 µg of a plasmid expressing a West Nile virus subgenomic replicon in which the structural genes have been replaced with green fluorescent protein(GFP) (23), and 3 µg of a plasmid expressing the WT or mutant C-prM-E structural genes of interest using Lipofectamine 3000 (Cat# L3000-015; ThermoFisher Scientific) according to manufacturer’s protocol. After 4 hours of incubation at 37°C, media was replaced and cells transferred to 30°C. Virus-containing supernatant was collected on days 3–6 post-transfection, pooled, passed through a 0.22 µm filter (Cat# SE1M179M6, Millipore-Sigma), aliquoted, and stored at −80°C. Mature RVP stocks were produced using the same protocol, except with the addition of 1 µg of a human furin-expressing plasmid (provided by Ted Pierson, NIH).

### Determining infectious titer of RVPs

Raji-DCSIGNR cells (2e5/well in 20 µL) were infected with 10 × 2-fold serial dilutions of an equal volume of each RVP stock in 384-well plates (Cat# 164688, ThermoFisher Scientific) and incubated at 37°C. Two days later, cells were fixed in a final concentration of 2% paraformaldehyde (Cat# 15714S; Electron Microscopy Sciences) and % GFP positive cells were enumerated by flow cytometry (Intellicyt iQue Screener PLUS, Sartorius AG). Infectious titers were determined from the most linear portions of the dose-response infectivity curves using the following formula: [(%GFP positive cells/100) * (number of cells infected)/volume of virus] * virus dilution factor.

### Production and purification of antibodies

Heavy and light variable region sequences of F25.S02 were obtained by single B cell transcriptomics as previously described (10). Variable region sequences for other bnAbs were determined from the following protein database IDs: 4UT9 (EDE1-C10), 4UTA (EDE1-C8), 4UT6 (EDE2-B7), 4UTB (EDE2-A11), and 7BUD (SIgN-3C). Corresponding cDNA sequences were codon-optimized, synthesized (Twist Bioscience, South San Francisco, CA), and cloned into mammalian expression vectors provided by Patrick Wilson (University of Chicago): AbVec-hIgG1 (GenBank accession # FJ475055) and AbVec-hIgKappa (GenBank accession # FJ475056) or AbVec-hIgLambda (GenBank accession # FJ517647). All AbVec antibody expression plasmids (IgG1-heavy, kappa, and lambda) were confirmed by Sanger sequencing using the primer “AbVec sense”: GCTTCGTTAGAACGCGGCTAC. Sequence-confirmed heavy and light chain plasmids were co-transfected into ExpiCHO-S cells at 0.8 ng/mL total DNA concentration at 1:1 mass ratio using OptiPro serum-free medium (Cat#12309, Gibco) and Expifectamine CHO Transfection Kit (Cat# A29130, Gibco) according to the manufacturer’s instructions. Eight days post-transfection, IgG-containing supernatant was collected, clarified by centrifugation (3,220 × *g* for 10 minutes), and filtered through a 0.45 µm membrane. IgG was purified using MabSelect Sure LC protein A agarose beads (Cat# 17-5474-01, Cytiva Life Sciences) according to the manufacturer’s instructions. Mouse monoclonal antibody 4G2 was purified from the hybridoma D1-4G2-4-15 (Cat# HB-112, ATCC) by the Fred Hutchinson Cancer Center Antibody Technology Core.

### Neutralization assays

Neutralization assays were performed in 384-well plates (Cat# 164688, ThermoFisher Scientific). RVPs were diluted to 5%–10% infectivity (20 µL/well) and incubated for 1 hour at room temperature with 10 × 5-fold serial dilutions of an equal volume of antibodies before infection of an equal volume of Raji-DCSIGNR cells (2e5/well) at 37°C for 2 days. Cells were fixed with 2% paraformaldehyde and % GFP positive cells were determined using flow cytometry (Intellicyt iQue Screener PLUS, Sartorius AG). Infection was normalized to conditions without antibody. The antibody concentration that neutralized 50% infection (IC50) was estimated by non-linear regression with a variable slope and the bottom and top of the curves constrained to 0% and 100%, respectively (GraphPad Prism 9.5.1).

### Determining E protein glycosylation status

RVP stocks prepared as described above were concentrated by microcentrifugation overnight (20,000 × *g* at 4°C) through a 20% sucrose cushion (0.25 mL sucrose per 1 mL supernatant). RVP pellets were resuspended in 5 mM HEPES, 150 mM NaCl, 0.1 mM EDTA (HNE buffer), pH adjusted to 7.4. For SDS-PAGE, concentrated RVPs were lysed by incubating at 55°C for 20 min with Bolt LDS sample buffer (Cat# B0007, ThermoFisher Scientific). E protein glycosylation status was determined by treating RVP lysates with PNGase F (Cat# P0704S, New England Biolabs) for 3 hours at 37°C. Treated and untreated RVP lysates were run on a 4%–12% Bis-Tris gel (Cat# NW04120BOX, ThermoFisher Scientific) for 1 hour at 100V, and transferred to a nitrocellulose membrane using the iBlot system (Cat# IB21001, ThermoFisher Scientific). Membranes were blocked for 1 hour at room temperature in blocking buffer (3% non-fat milk in 25 mM Tris, 0.15 M NaCl, 0.05% Tween 20 at pH 7.5 [TBS-T]), followed by overnight incubation with rocking at 4°C with 3 µg/mL mouse primary anti-E antibody, 4G2 (purified from hybridoma as described above), or ZV-67 (a gift from Michael Diamond, Washington University, St. Louis) to detect E protein of DENV2 or ZIKV, respectively. Primary antibodies were diluted in blocking buffer. The next day, membranes were washed three times with TBS-T, followed by incubation for 1 hour at room temperature with a horseradish peroxidase conjugated anti-mouse secondary antibody (Cat# NA931-1ML, Millipore Sigma) diluted 1:1,000 in blocking buffer. Membranes were again washed three times with TBS-T and protein bands detected using chemiluminescence (Cat# RPN3004, Cytiva Life Sciences).

### Statistical analysis

Ratio paired *t*-tests (Fig. 1H) or Dunnett’s multiple comparisons test (Fig. 2G) were performed using GraphPad Prism version 10.1.0.

## SUPPLEMENTAL MATERIAL

Supplemental Figures mbio.03048-23-s0001.docx Fig. S1 and S2.

ASM does not own the copyrights to Supplemental Material that may be linked to, or accessed through, an article. The authors have granted ASM a non-exclusive, world-wide license to publish the Supplemental Material files. Please contact the corresponding author directly for reuse.
