# Supplementary material for: Defining the impact of flavivirus envelope protein glycosylation site mutations on sensitivity to broadly neutralizing antibodies
Source: mBio. 2024 Jan 9;15(2):e03048-23. doi: 10.1128/mbio.03048-23 (PMC10865826; doi:10.1128/mbio.03048-23)
Supplement: Supplemental Figures — Fig. S1 and S2. [file mbio.03048-23-s0001.docx]

**SUPPLEMENTAL INFO**

**
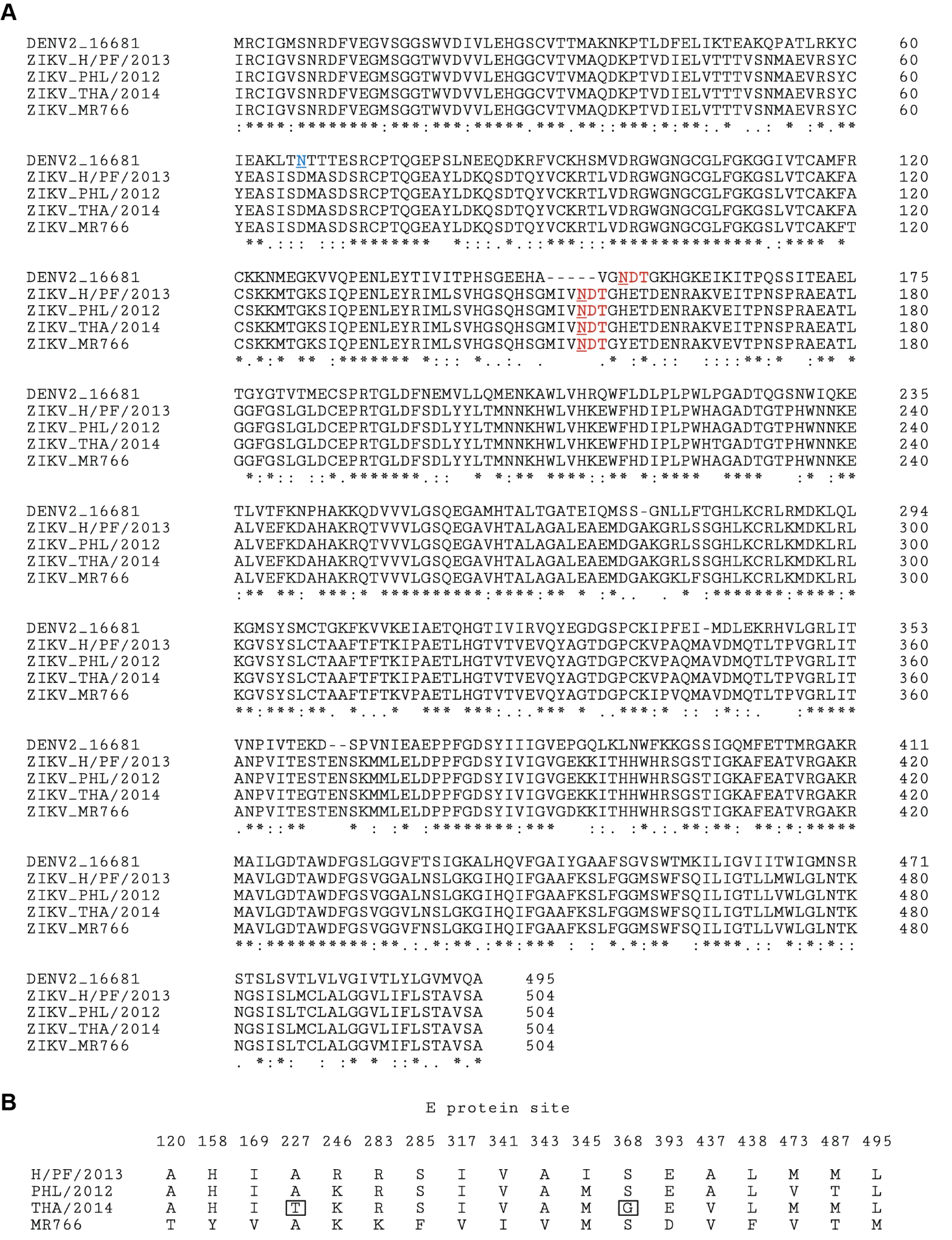
**

**Figure S1. E protein sequence alignment of ZIKV and DENV. (A)** Multiple sequence alignment of the indicated DENV2 and ZIKV E protein sequences created using Clustal Omega tool (EMBL-EBI) [(24)](https://paperpile.com/c/VnBGvz/PvBlg). Genbank Genome Accession numbers are as follows: NC_001474.2 (DENV2_16681), KJ776791.2 (ZIKV_H/PF/2013), KU681082.3 (ZIKV_PHL/2012), KU681081.3 (ZIKV_THA/2014), HQ234498.1 (ZIKV_MR766). The PNGS motif in the 150 loop is shown in red. Within this motif, site N153 or N154 in DENV2 or ZIKV, respectively, is underlined. The additional PNGS at DENV2 residue N67 is shown in blue and underlined. Under the alignment, ‘*’ indicates completely conserved residue; ‘:’ indicates amino acids with strongly similar properties (score of >0.5 in the Gonnet PAM 250 matrix) and ‘.’ indicates amino acids with weakly similar properties (score of <=0.5 in the Gonnet PAM 250 matrix). **(B)** E protein sites of amino acid variation among the ZIKV strains tested in this study (Genbank Genome Accession numbers as in (A)). E protein sites and amino acid residues that distinguish ZIKV strain THA/2014 from the others are boxed.

**
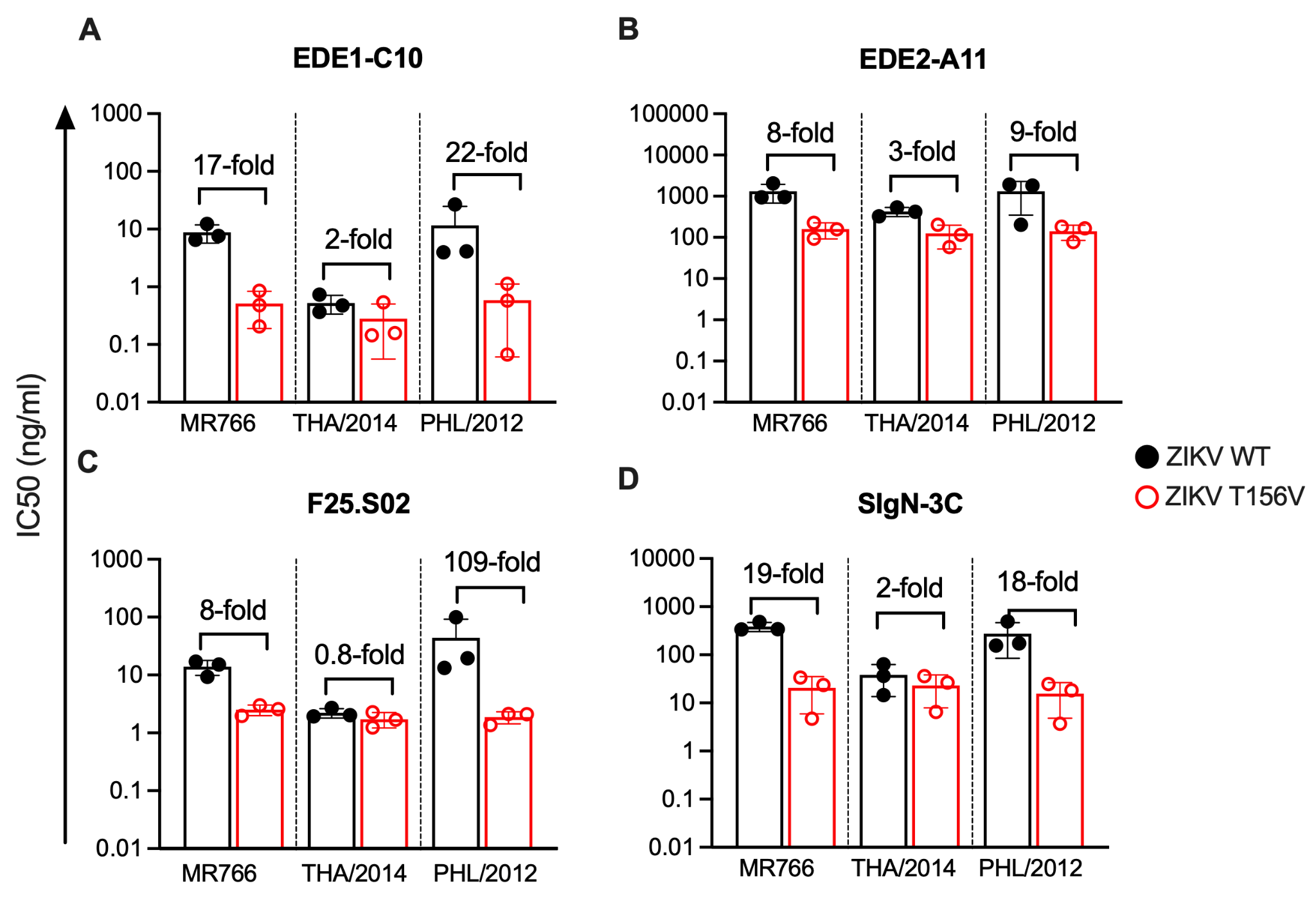
**

**Figure S2. Effect of T156V mutation on sensitivity of additional ZIKV strains to neutralizing antibodies**

**(A-E)** Bar graphs show mean IC50 values of the indicated antibodies against WT (black) or T156V (red) RVPs of ZIKV strains shown on the x-axes from three independent experiments, each represented by a data point. Error bars indicate the standard deviation.

**REFERENCE**

24. [Sievers F, Wilm A, Dineen D, Gibson TJ, Karplus K, Li W, Lopez R, McWilliam H, Remmert M, Söding J, Thompson JD, Higgins DG. 2011. Fast, scalable generation of high-quality protein multiple sequence alignments using Clustal Omega. Mol Syst Biol 7:539.](http://paperpile.com/b/VnBGvz/PvBlg)
